# C1 posterior arch screw as an auxiliary anchor in posterior reconstruction for atlantoaxial dislocation associated with type II odontoid fracture: a case report and review of the literature

**DOI:** 10.1186/2193-1801-3-672

**Published:** 2014-11-13

**Authors:** Narihito Nagoshi, Kota Suda, Tomonori Morita, Satoko Matsumoto, Seiji Iimoto, Keigo Yasui, Miki Komatsu, Yosuke Kobayashi, Akio Minami, Yoshiaki Toyama, Morio Matsumoto, Masaya Nakamura

**Affiliations:** Department of Orthopedic Surgery, Spinal Cord Injury Center, Hokkaido Chuo Rosai Hospital, 3-1, Higashi-yonjo Minami-icchome, Bibai, 072-0015 Japan; Department of Orthopedic Surgery, Keio University School of Medicine, 35 Shinanomachi, Tokyo, 160-8582 Shinjuku-ku Japan

**Keywords:** Posterior arch screw, Atlantoaxial dislocation, Odontoid fracture, Vertebral artery injury

## Abstract

**Introduction:**

Although pedicle or lateral mass screws are usually chosen to fix atlantoaxial (C1-C2) instability, there is an increased risk for vertebral artery (VA) injury when used in patients with bone or arterial anomalies or osteoporotic bone. Here we report the C1 posterior arch screw as a new technique for upper cervical fixation.

**Case description:**

A 90-year-old man complained of upper cervical pain after falling in his house. The initial computed tomography (CT) scan showed C1-C2 posterior dislocation with a type II odontoid fracture. The patient underwent C2 fracture reduction and posterior C1-C2 fixation. On the right side of C1, because lateral mass screw placement could cause injury to the dominant VA considering a risk in oldest-old osteoporotic patients, a posterior arch screw was chosen instead as an auxiliary anchor. An intralaminar screw was placed on the right side of C2 because a high-riding VA was observed. A lateral mass screw and a pars interarticularis screw were placed on the left side of C1 and C2, respectively. Ten months later, the odontoid fracture had healed, with normal anatomical alignment. Although the patient experienced slight weakness when spreading his bilateral fingers, his overall condition was good.

**Discussion and evaluation:**

We have presented a novel technique using C1 posterior arch screws for the fixation of a C1-C2 dislocation. Such a screw is an alternative to the C1 lateral mass screw in patients who are at risk for a VA injury because of anomalous bone and arterial structures or poor bone quality.

**Conclusions:**

Although there have been few comparable studies, and the long-term outcome is unknown, fixation with a posterior arch screw could be a beneficial choice for surgeries involving the upper cervical region.

## Introduction

Instability in the atlantoaxial (C1-C2) complex can arise from trauma, malignancy, inflammatory disease, or congenital malformation. To remedy this pathological condition, surgical intervention is often needed to achieve realignment and fixation of the vertebrae. The methods used for C1 posterior fixation have included posterior wiring, transarticular screws, and pedicle or lateral mass screws (Harms & Melcher 2001; Goel & Laheri 1994; Brooks & Jenkins 1978). Recently, the use of wiring or hooks has been replaced by screw fixation, which provides rigidity and prevents most instances of postoperative movement (Henriques et al. 2000). However, screw fixation is associated with an increased risk for vertebral artery (VA) injury in patients whose VA is found in an anomalous location, or who display abnormal bone morphology or have osteoporosis. To avoid the VA injury, various techniques have been reported. If the VA is in an anomalous location, screw purchase at the C1 superior lateral mass may be used, instead of C1-C2 transarticular screws (Hong et al. 2011; Yamazaki et al. 2012). If an anomalous VA across the posterior surface of the C1 lateral mass can be mobilized inferiorly, a screw may be inserted from the inferior lateral mass (Umebayashi et al. 2013). Another technique is to skip the C1 screw purchase and to extend the fixation range rostrally and caudally (Yamazaki et al. 2012). With each method, careful preoperative assessment of the VA courses is important in planning the fixation.

In this report, we present the case of an elderly patient who sustained a primary atlantoaxial dislocation associated with a type II odontoid fracture that required the use of a C1 posterior arch screw.

## Case report

**Figure 1:**
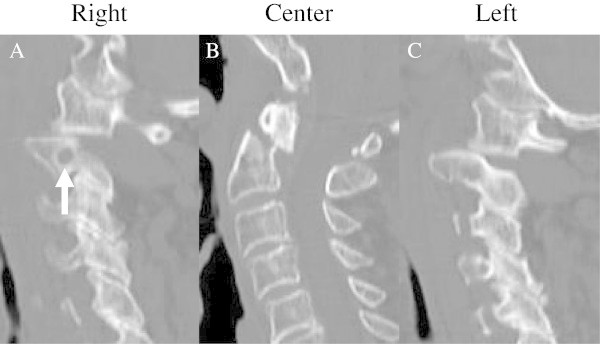
**Sagittal computed tomography (CT) reconstruction images before surgery.** Posteriorly displaced odontoid fracture **(B)** and bilateral dislocation of the C1-C2 facet joint **(A and** **C)** were prominent. To the right of C2, a high-riding vertebral artery (VA) was revealed (**A**; arrow).

A 90-year-old man was transferred from a regional emergency department, having stumbled and fallen in his house. The patient complained of upper cervical pain. A neurological examination revealed a slight weakness in the muscle strength of his upper extremities. The sensations to light touch and a pin prick were intact, however. Cervical spine radiography and computed tomography (CT) scans revealed that C2 was fractured at the odontoid process, with 6 mm of displacement (Figure 1B). This was a type II fracture based on the Anderson and D’ Alonzo classification (Anderson & D’Alonzo 1974). C1 was displaced posteriorly relative to C2, with bilateral displacement of the lateral joints (Figures 1A and C). CT and magnetic resonance (MR) angiography revealed that the right-side VA was dominant. Conservative treatment consisting of reduction and fixation with a halo vest was tried initially. However, because complete reduction of the C1-C2 dislocation was not achieved, we decided to perform surgery to reduce the dislocation and to fix the C1-C2 joint.

**Figure 2:**
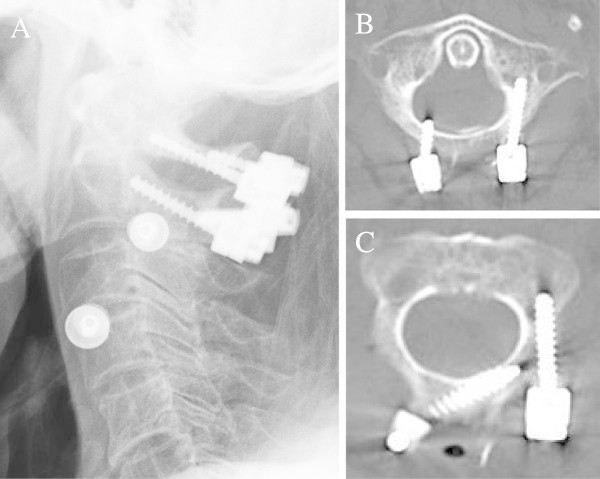
**X-ray and computed tomography (CT) images after surgery.** Radiography revealed posterior fixation and reduced C1-C2 dislocation **(A)**. Axial CT images showed posterior arch screw and lateral mass screw at C1 **(B)**, and intralaminar screw and pars interarticularis screw at C2 **(C)**.

The patient was given general anesthesia and treated in the prone position. The odontoid fracture was reduced successfully back into the correct anatomic position through a combination of gentle manual traction and neck flexion, using a fluoroscope for guidance. Next, using standard anatomical landmarks, a unicortical lateral mass screw was placed via the posterior arch on the left side of C1, as advocated by Tan et al. (Figure 2A and B) (Tan et al. 2003). On the left side at C2, a pars interarticularis screw was placed (Figure 2A and C). Since a high-riding VA was seen on the right side of C2, a unicortical intralaminar screw was placed there (Figure 2C). On the right side of C1, we chose a posterior arch screw, because a lateral mass screw placed at that location could put the oldest-old osteoporotic patient at risk for a dominant VA injury if the screw ever loosened (Figure 2A and B). We first drilled 3-mm bicortical pilot holes at an entry point approximately 1 cm lateral to the midline. To prevent injury to the underlying dura mater, a spatula was placed at the exit point of the drill, between the anterior side of the posterior arch and the dura mater. The holes were then carefully tapped to a diameter of 3.5 mm to prevent splitting of the arch when the screw was inserted. A 3.5-mm polyaxial screw of 12-mm length (Medtronic Inc.) was next placed at C1 (Figure 2B). After securing the titanium rods, decortication and local bone graft were performed. Fluoroscopy was used intraoperatively to confirm the correct placement of the hardware and the anatomic alignment.

**Figure 3:**
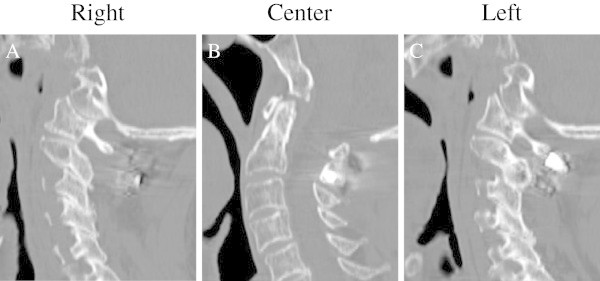
**Sagittal computer tomography reconstruction images three months after the surgery.** The fracture was healed **(B)**, and the C1-C2 reduction was maintained **(A, C)**.

Postoperatively, the patient was immobilized in a hard cervical collar. Three months after the surgery, the odontoid fracture had healed into an anatomically correct alignment, and the cervical collar was removed (Figure 3A-C). Ten months later, the patient was in good condition, showing only a slight weakness when trying to abduct his bilateral fingers.

Written informed consent was obtained from the patient for publication of this case report and any accompanying images.

## Discussion

Recently, the preferred anchor screw for C1 stabilization has been the lateral mass screw. In this case, however, the patient was oldest-old, with low bone quality, which increased the chance of the screws loosening after surgical fixation. In fact, previous reports have demonstrated that a lower bone mineral density is strongly associated with a lower screw pullout strength (Savage et al. 2011). Therefore, we assumed that the placement of a C1 lateral mass screw on the dominant side of the VA would not be safe, especially for the osteoporotic patient. On the same side of C2, intralaminar screw was purchased to avoid the VA injury because the intralaminar screw could achieve similar biomechanical stability to C2 pedicle screw (Savage et al. 2011). On the left side of C1, we placed a unicortical lateral mass screw via the posterior arch. Biomechanically, this screw has a similar pullout strength as a bicortical C1 lateral mass screw inserted just under the posterior arch (Ma et al. 2009). The unicortical screw prevents injury to the hypoglossal nerve and internal carotid artery, which are located on the anterior surface of the lateral mass of C1 (Zarro et al. 2013). Thus, here we placed the posterior arch screw on the right side as an anchor screw to augment the C1-C2 stability, since optimal positioning and sufficient fixation strengths were achieved for the C1 and C2 screws on the opposite side.

Some key techniques for placing the posterior arch screws are noteworthy. First, we used a 3.5-mm tap for a 3.5-mm screw. It is possible that a smaller tap would have provided an improved screw purchase. However, because the bone quality was weak, we believed that the excessive torque created during screw insertion could lead to splitting and fracturing of the posterior arch. Therefore, we used the same size tap as the screw diameter. The next consideration was the entry point of the screw, since the ease in securing the rods depends on the position of the connected screws. In this case, we took into account the position of the C2 intralaminar screw head, and placed the posterior arch screw about 10 mm from the midline of the posterior tubercle. The exit point for the screws is another important consideration, because a longer screw could increase the fixation strength. However, if the screw was inserted in the wrong direction, there still existed the risk of VA injury. To put the screw properly, we rigorously examined the VA courses by preoperative CT angiography, and purchased the screw with sufficient gap between the tip of the screw and the artery. Moreover, when calibrating in the CT images, height of the posterior arch in the inserted place was 9.0 mm. Since the diameter of the screw was 3.5 mm, we judged the screw purchase was safe if inserted in the middle of the arch. Thus, thorough preoperative planning and careful intraoperative technique are needed for the successful placement of screws.

There are some risks associated with posterior arch screws. During surgery, the posterior arch of the atlas can be destroyed if its height or thickness is insufficient for the screw purchase. In addition, deep screw purchase can result in dural tear or spinal cord injury, requiring the use of a spatula to protect these tissues. After surgery, screw loosening can occur due to biomechanical weakness. Therefore, a posterior arch screw should be used only in a complementary role for C1-2 fixation, and the indication for this placement is quite limited.

A review of the literature revealed only one report describing the use of posterior arch screws in the treatment of cervical fracture (Carmody et al. 2010). In that case, the patient was diagnosed with a type II odontoid fracture, and a CT angiogram uncovered bilateral persistent first intersegmental arteries, which were anomalous VAs running through the C1-C2 neural foramina (Carmody et al. 2010). Because the anomalous VA courses precluded the placement of C1 lateral mass screws, bicortical posterior arch screws were placed instead. In that case, the posterior arch screws were placed bilaterally as main anchors for the atlas. In contrast, in our case the posterior arch screw was placed unilaterally, to augment the fixation by contralateral anchors consisting of a C1 lateral mass screw and C2 pars interarticularis screw. The use of posterior arch screws as major anchors in osteoporotic patients could result in screw pullout and pseudoarthrosis, and should be avoided.

There have been a few reports describing the biomechanical testing of posterior arch screw fixations that are slightly different from the technique discussed here. Zarro et al. compared the pullout strength of C1 lateral mass screws versus unicortical posterior arch screws, and demonstrated that the posterior arch screws provided significantly stronger resistance to pullout via axial load than did the lateral mass screws (Zarro et al. 2013). Jin et al. examined the range of cervical motion after C1-C2 fixation using a C1 unicortical posterior arch screw or a lateral mass screw, and found no significant differences between the screws in terms of flexibility values, including flexion-extension or rotation (Jin et al. 2013). Although additional studies examining the biomechanical properties of bicortical posterior arch screws are needed, the results of these previous reports indicate that C1 posterior arch screw fixation constitutes an alternative method for C1-C2 fixation.

Traumatic C1-C2 dislocation complicating an odontoid fracture is relatively rare, and most of these cases are probably fatal (Pissonnier et al. 2013). Some previous reports showed that reduction and fixation could be achieved by halo vest, resulting in successful healing of the dislocation and fracture (Spoor et al. 2008; Oh et al. 2010). However, similar to the present case, in most of the cases described in the literature, the use of a halo vest alone failed to reduce the dislocation, and therefore surgical fixation was performed secondarily (Lenehan et al. 2010; Hopf et al. 2009; Moreau et al. 2012; Przybylski & Welch 1996). Bransford et al. demonstrated that the main reason for the failure of halo vest immobilization was instability caused by cervical spine injuries, which included odontoid fracture (Bransford et al. 2009). Therefore, surgical fixation should be considered for the treatment of a C1-C2 dislocation associated with an odontoid fracture, even if external fixation by halo vest is initially performed. Indeed, some authors claim that surgical fixation should be the first step in treating the instability of a C1-C2 dislocation with an associated odontoid fracture (Pissonnier et al. 2013; Goel et al. 2010). Regarding surgical treatments, most patients with a C1-C2 dislocation have been treated by C1-C2 posterior fixation including lateral mass and pedicle screws, or transarticular screws (Lenehan et al. 2010; Hopf et al. 2009; Goel et al. 2010; Fuentes et al. 2001). In some cases, when the axis fracture was comminuted or when the C1-C2 joint line could not be clearly identified by fluoroscopy during surgery, the range of fixation was extended rostrally and caudally (Moreau et al. 2012; Przybylski & Welch 1996). In a recent report, anterior fixation was performed using bilateral screws through lateral C1-C2 articulations (Riouallon & Pascal-Moussellard 2014). The optimal surgical technique should be selected based on the pathological conditions.

## Conclusions

We have presented a novel technique using C1 posterior arch screws for the fixation of a C1-C2 dislocation. Such a screw is an alternative to the C1 lateral mass screw in patients who are at risk for a VA injury because of anomalous bone structures or poor bone quality. Although there have been few comparable studies, and long-term outcome or biomechanical testing research has yet to be described, fixation with a posterior arch screw could be a reasonable treatment option for surgeries of the upper cervical region.

## References

[CR1] Anderson LD, D’Alonzo RT (1974). Fractures of the odontoid process of the axis. J Bone Joint Surg Am.

[CR2] Bransford RJ, Stevens DW, Uyeji S, Bellabarba C, Chapman JR (2009). Halo vest treatment of cervical spine injuries: a success and survivorship analysis. Spine.

[CR3] Brooks AL, Jenkins EB (1978). Atlanto-axial arthrodesis by the wedge compression method. J Bone Joint Surg Am.

[CR4] Carmody MA, Martin MD, Wolfla CE (2010). Persistent first intersegmental vertebral artery in association with type II odontoid fracture: surgical treatment utilizing a novel C1 posterior arch screw: case report. Neurosurgery.

[CR5] Fuentes S, Bouillot P, Palombi O, Ducolombier A, Desgeorges M (2001). Traumatic atlantoaxial rotatory dislocation with odontoid fracture: case report and review. Spine.

[CR6] Goel A, Laheri V (1994). Plate and screw fixation for atlanto-axial subluxation. Acta Neurochir.

[CR7] Goel A, Figueiredo A, Maheshwari S, Shah A (2010). Atlantoaxial manual realignment in a patient with traumatic atlantoaxial joint disruption. J Clin Neurosci: Offic J Neurosurg Soc Australasia.

[CR8] Harms J, Melcher RP (2001). Posterior C1-C2 fusion with polyaxial screw and rod fixation. Spine.

[CR9] Henriques T, Cunningham BW, Olerud C, Shimamoto N, Lee GA, Larsson S, McAfee PA (2000). Biomechanical comparison of five different atlantoaxial posterior fixation techniques. Spine.

[CR10] Hong JT, Jang WY, Kim IS, Yang SH, Sung JH, Son BC, Lee SW (2011). Posterior C1 stabilization using superior lateral mass as an entry point in a case with vertebral artery anomaly: technical case report. Neurosurgery.

[CR11] Hopf S, Buchalla R, Elhoft H, Rubarth O, Borm W (2009). [Atypical dislocated dens fracture type II with rotational atlantoaxial luxation after a riding accident]. Unfallchirurg.

[CR12] Jin GX, Wang H, Li L, Cui SQ, Duan JZ (2013). C1 posterior arch crossing screw fixation for atlantoaxial joint instability. Spine.

[CR13] Lenehan B, Guerin S, Street J, Poynton A (2010). Lateral C1-C2 dislocation complicating a type II odontoid fracture. J Clin Neurosci: Offic J Neurosurg Soc Australasia.

[CR14] Ma XY, Yin QS, Wu ZH, Xia H, Liu JF, Xiang M, Zhao WD, Zhong SZ (2009). C1 pedicle screws versus C1 lateral mass screws: comparisons of pullout strengths and biomechanical stabilities. Spine.

[CR15] Moreau PE, Nguyen V, Atallah A, Kassab G, Thiong’o MW, Laporte C (2012). Traumatic atlantoaxial dislocation with odontoid fracture: a case report. Orthop Traumatol Surg Res: OTSR.

[CR16] Oh JY, Chough CK, Cho CB, Park HK (2010). Traumatic atlantoaxial rotatory fixation with accompanying odontoid and c2 articular facet fracture. J Korean Neurosurg Soc.

[CR17] Pissonnier ML, Lazennec JY, Renoux J, Rousseau MA (2013). Trauma of the upper cervical spine: focus on vertical atlantoaxial dislocation. Eur Spine J: Offic Publ Eur Spine Soc Eur Spinal Deformity Soc Eur Section Cervical Spine Res Soc.

[CR18] Przybylski GJ, Welch WC (1996). Longitudinal atlantoaxial dislocation with type III odontoid fracture. Case report and review of the literature. J Neurosurg.

[CR19] Riouallon G, Pascal-Moussellard H (2014). Atlanto-axial dislocation complicating a type II odontoid fracture. Reduction and final fixation. Orthop Traumatol Surg Res: OTSR.

[CR20] Savage JW, Limthongkul W, Park HS, Zhang LQ, Karaikovic EE (2011). A comparison of biomechanical stability and pullout strength of two C1-C2 fixation constructs. Spine J: Offic J North Am Spine Soc.

[CR21] Spoor AB, Diekerhof CH, Bonnet M, Oner FC (2008). Traumatic complex dislocation of the atlanto-axial joint with odontoid and C2 superior articular facet fracture. Spine.

[CR22] Tan M, Wang H, Wang Y, Zhang G, Yi P, Li Z, Wei H, Yang F (2003). Morphometric evaluation of screw fixation in atlas via posterior arch and lateral mass. Spine.

[CR23] Umebayashi D, Hara M, Nakajima Y, Nishimura Y, Wakabayashi T (2013). Posterior fixation for atlantoaxial subluxation in a case with complex anomaly of persistent first intersegmental artery and assimilation in the C1 vertebra. Neurol Med Chir.

[CR24] Yamazaki M, Okawa A, Furuya T, Sakuma T, Takahashi H, Kato K, Fujiyoshi T, Mannoji C, Takahashi K, Koda M (2012). Anomalous vertebral arteries in the extra- and intraosseous regions of the craniovertebral junction visualized by 3-dimensional computed tomographic angiography: analysis of 100 consecutive surgical cases and review of the literature. Spine.

[CR25] Zarro CM, Ludwig SC, Hsieh AH, Seal CN, Gelb DE (2013). Biomechanical comparison of the pullout strengths of C1 lateral mass screws and C1 posterior arch screws. Spine J.

